# Recent Advances in Aptasensors For Rapid and Sensitive Detection of *Staphylococcus Aureus*

**DOI:** 10.3389/fbioe.2022.889431

**Published:** 2022-05-23

**Authors:** Wei Chen, Qingteng Lai, Yanke Zhang, Zhengchun Liu

**Affiliations:** ^1^ Department of Clinical Laboratory, Xiangya Hospital of Central South University, Changsha, China; ^2^ National Clinical Research Center for Geriatric Diseases, Xiangya Hospital of Central South University, Changsha, China; ^3^ Hunan Key Laboratory for Super Microstructure and Ultrafast Process, School of Physics and Electronics, Central South University, Changsha, China; ^4^ Department of Microbiology, School of Basic Medical Science, Central South University, Changsha, China

**Keywords:** *Staphylococcus aureus*, aptasensor, optical biosensor, electrochemical biosensor, nanomaterials, POCT

## Abstract

The infection of *Staphylococcus aureus (S.aureus)* and the spread of drug-resistant bacteria pose a serious threat to global public health. Therefore, timely, rapid and accurate detection of *S. aureus* is of great significance for food safety, environmental monitoring, clinical diagnosis and treatment, and prevention of drug-resistant bacteria dissemination. Traditional *S. aureus* detection methods such as culture identification, ELISA, PCR, MALDI-TOF-MS and sequencing, etc., have good sensitivity and specificity, but they are complex to operate, requiring professionals and expensive and complex machines. Therefore, it is still challenging to develop a fast, simple, low-cost, specific and sensitive *S. aureus* detection method. Recent studies have demonstrated that fast, specific, low-cost, low sample volume, automated, and portable aptasensors have been widely used for *S. aureus* detection and have been proposed as the most attractive alternatives to their traditional detection methods. In this review, recent advances of aptasensors based on different transducer (optical and electrochemical) for *S. aureus* detection have been discussed in details. Furthermore, the applications of aptasensors in point-of-care testing (POCT) have also been discussed. More and more aptasensors are combined with nanomaterials as efficient transducers and amplifiers, which appears to be the development trend in aptasensors. Finally, some significant challenges for the development and application of aptasensors are outlined.

## Introduction

*S.aureus* is one of the most pathogenic pathogens in the world and it was first discovered and named by Dr. Alexander Ogston in 1880 (Ogston, 1882). *S. aureus* is a major cause of foodborne poisoning (Hulme, 2017), with more than 240,000 cases annually in the United States, and it is a major public health concern (Zhang et al., 2019). *S. aureus* is also the most common pathogen in purulent infections in humans, and can enter any organ or enter the bloodstream when the host immunity is weakened or the skin and mucosal barriers are disrupted. It can cause skin and soft tissue infections (impetigo, folliculitis and scalded skin syndrome), and severe systemic diseases such as bacteremia, endocarditis, osteomyelitis, hemolytic pneumonitis, and toxic shock syndrome (Tong et al., 2015). Mild skin and mucosal infections are usually self-limiting, while severe systemic infections are usually associated with high mortality (20∼50%), high recurrence rate (5∼10%), and persistent injury (more than three points out of the survivors) (Kern and Rieg, 2020). Methicillin-resistant *S. aureus* (MRSA) has been a serious threat to global public health since it was first described in 1961 (Jevons, 1961). About 40∼60% of nosocomial *S. aureus* infections are MRSA in developed countries such as the United States, Europe and Japan (Fluit et al., 2001), and the MRSA infection rate is higher in developing countries (>70%), probably due to the widespread and improper use of antibiotics, and the spread of drug-resistant bacteria (Chen and Huang, 2014).

The rapid and accurate detection of *S. aureus* is of great significance to food safety, environmental monitoring, clinical diagnosis and treatment, and prevention of the spread of drug-resistant bacteria. The gold standard for detecting *S. aureus* is still culture identification, the results are accurate and reliable, economical and simple, but it is very time-consuming, usually taking 1∼2 days to form visible colonies on agar plates, and then 1∼2 days for biochemical identification and serological typing. In recent years, several rapid and automated detection methods have been developed, such as enzyme-linked immunosorbent assay (ELISA) (Nouri et al., 2018), polymerase chain reaction (PCR) (Umesha and Manukumar, 2018), next-generation sequencing and matrix-assisted laser desorption/ionization time-of-flight mass spectrometry (MALDI-TOF-MS) (Gill et al., 2019; Rajapaksha et al., 2019), et al. But they are complex to operate, requiring professionals and expensive and complex machines. Therefore, it is still a hot issue to develop a fast, simple, low-cost, specific and sensitive *S. aureus* detection method.

In the past two decades, biosensors have been more and more widely used in pathogen detection due to fast, low cost, high sensitivity, low sample volume, automation and portability, and have been proposed as the most promising alternative to traditional pathogen detection methods (Furst and Francis, 2019; Cesewski and Johnson, 2020). The biosensor is mainly composed of three parts: bioreceptor, transducer and signal readout system (Yu et al., 2021). Biorecognition element refers to the “biological receptor” that can recognize the target with strong affinity and high specificity, and is the most critical part of the biosensor, which determines the specificity (selectivity) of the biosensor (Morales and Halpern, 2018). Antibodies, peptides and bacteriophages are the most commonly used biorecognition elements, among which antibodies are considered to be the gold standard recognition element in biosensors due to their high affinity and specificity between antibodies and antigens (Sharma et al., 2016). In the past few decades, antibody technology has developed rapidly and has made great contributions to the development of medicine and the entire life sciences. However, there are still many limitations in the application of antibodies such as the antibody development process is cumbersome and complicated and it is very challenging to generate antibodies that recognize small molecule targets (Zhou and Rossi, 2017; Liu et al., 2021a). Aptamers are another class of biorecognition elements that have attracted great interest in biosensing in recent years, and are known as “chemical antibodies” due to their ability to interact with their targets with strong affinity and high specificity similar to antigen-antibody interactions (Banerjee, 2010).

**FIGURE 1:**
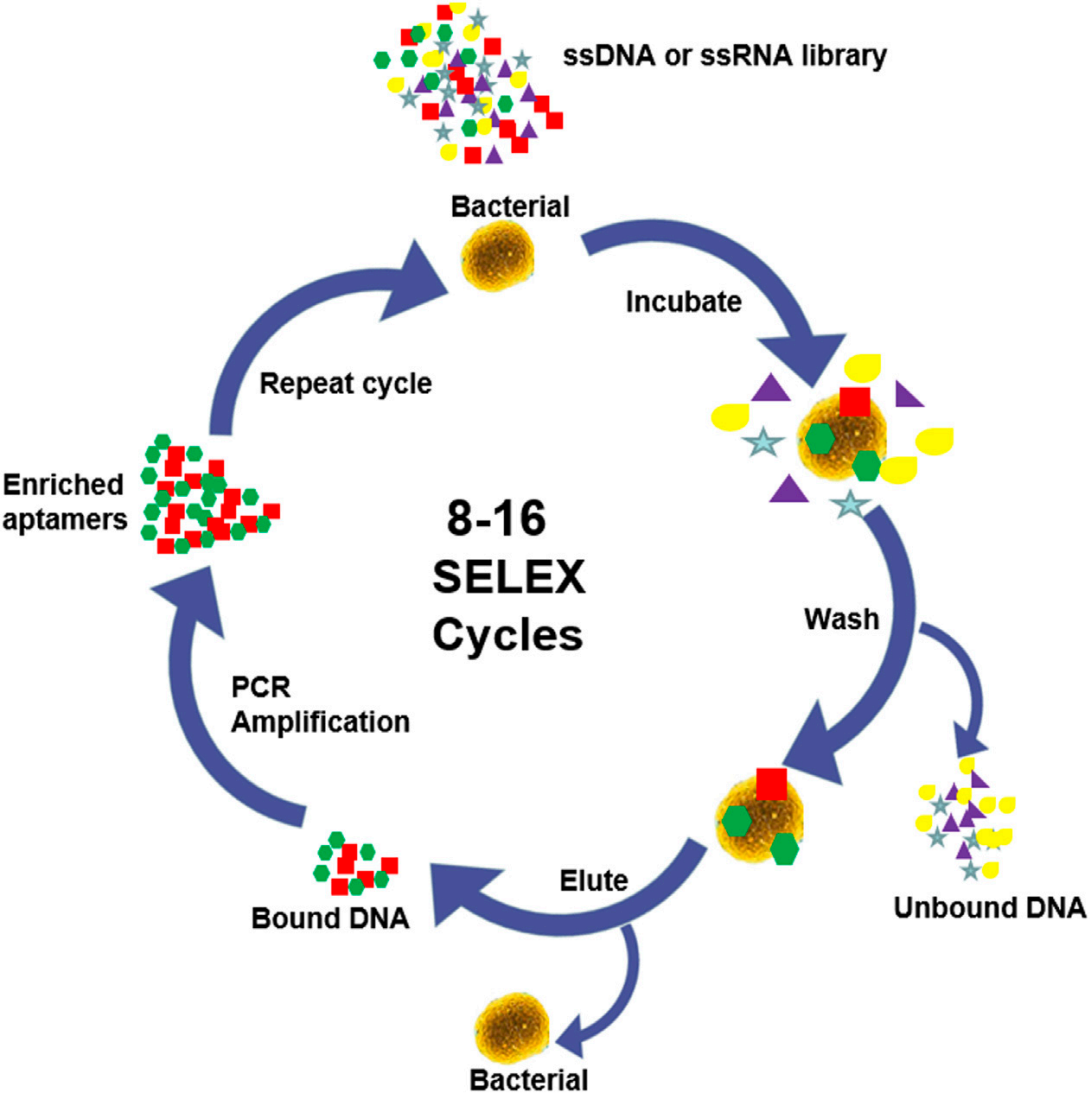
SELEX process.

Aptamer is a specific oligonucleotide sequence (most commonly single-stranded DNA or RNA), usually ranging in length from 25 to 90 bases, and their binding to the target is based on the diversity of single-stranded nucleic acid structure and spatial conformation (Tok et al., 2000). In the presence of the target, aptamers pass through the pairing and electrostatic interactions between complementary bases in the chain, and self-adaptive folding into different types of secondary structures, including stems (Tok et al., 2000), inner loops, purine-rich bulges, hairpins, pseudoknots (Tuerk et al., 1992), kissing complexes (Boiziau et al., 1999), and G-quadruplex (Bock et al., 1992). Subsequently, these secondary structures assemble to form unique three-dimensional (3D) structures, resulting in strong affinity and high specificity for binding to target. Aptamers can efficiently and specifically attach to a wide range of targets, including small molecules, ions, peptides, proteins, viruses, bacteria and even cells (Shen et al., 2014). The affinity dissociation constants (Kd) of the aptamers with their cognate ligand are generally 1 pM∼1 mM, most of them are between 1 and 10 nM, which is much larger than that of the related non-cognate ligand, indicating that aptamers have good specificity (Alizadeh et al., 2017). RNA-type oligonucleotide aptamers were fist found by Tuerk and Ellington in 1990 by phylogenetic evolution of ligands with exponential enrichment (SELEX) (Ellington and Szostak, 1990; Tuerk and Gold, 1990). Conventional SELEX consists of multiple cycles, each cycle including 5 main steps as shown in Figure 1: synthesis, binding, isolation, elution and amplification (Hamula et al., 2011; Teng et al., 2016). Since conventional SELEX is a very time-consuming process, many methods have been introduced in the past few decades to generate higher efficiency and reliability aptamers, such as Cell-SELEX (Dwivedi et al., 2013; Amraee et al., 2017; Li et al., 2018; He et al., 2019), Genomic SELEX (Lorenz et al., 2006), IP-SELEX (Wang et al., 2013), Capture-SELEX (Reinemann et al., 2016), CE-SELEX (Mendonsa and Bowser, 2004; Berezovski et al., 2006a; Berezovski et al., 2006b), M-SELEX (Lou et al., 2009), AFM-SELEX (Miyachi et al., 2010), AEGIS-SELEX (Sefah et al., 2014), Animal-SELEX (Cheng et al., 2013a), etc.

Aptamer-based biosensors are widely used in the rapid detection of *S. aureus* due to their fast, specific, low cost, low sample volume, automated, and portable. The commonly used aptamer sequences against *S. aureus* by SELEX technique are shown in Table 1. Therefore, this paper focuses on the latest progress of aptasensors based on different transducer (optical and electrochemical) for *S. aureus* detection. The detection principle, linear range, sensitivity and detection time of aptasensors are presented in Table 2. Furthermore, the applications of aptasensors in POCT have also been discussed. This review also highlights that more and more nanomaterials have been used as immobilized recognition elements or amplified detection signals to improve the sensitivity of aptasensors. Finally, some significant challenges for the development and application of aptasensors are outlined.

**TABLE 1:** Aptamers selected against *S.aureus* by SELEX technique.

Aptamer name	Aptamer sequence (5–3′)	Kd (nM)	Ref
SA20	GCG​CCC​TCT​CAC​GTG​GCA​TCA​GAG​TGC​CGG​AAG​TTC​TGC​GTT​AT	70.86 ± 39.22	Cao et al. (2009)
SA23	GGG​CTG​GCC​AGA​TCA​GAC​CCC​GGA​TGA​TCA​TCC​TTG​TGA​GAA​CCA	61.50 ± 22.43	
SA31	TCC​CAC​GAT​CTC​ATT​AGT​CTG​TGG​ATA​AGC​GTG​GGA​CGT​CTA​TGA	82.86 ± 33.20	
SA34	CAC​AGT​CAC​TCA​GAC​GGC​CGC​TAT​TGT​TGC​CAG​ATT​GCC​TTT​GGC	72.42 ± 35.23	
SA43	TCG​GCA​CGT​TCT​CAG​TAG​CGC​TCG​CTG​GTC​ATC​CCA​CAG​CTA​CGT​C	210.70 ± 135.91	
SA17	TCC​CTA​CGG​CGC​TAA​CCC​CCC​CAG​TCC​GTC​CTC​CCA​GCC​TCA​CAC​CGC​CAC​CGT​GCT​ACA​AC	35.0	Chang et al. (2013)
SA61	TCC​CTA​CGG​CGC​TAA​CCT​CCC​AAC​CGC​TCC​ACC​CTG​CCT​CCG​CCT​CGC​CAC​CGT​GCT​ACA​AC	129.0	
A14	CAC​ACC​GCA​GCA​GTG​GGA​ACG​TTT​CAG​CCA​TGC​AAG​CAT​CAC​GCC​CGT	3.49 ± 1.43	Moon et al. (2015)
RAB1	CGG​GTG​GGC​TCC​AAT​ATG​AAT​CGC​TTG​CCC​TGA​CGC​TAT​CT	56 ± 87	Ramlal et al. (2018)
RAB3	CGT​AGT​CTA​GTG​TCG​ATT​AGT​TTC​CTT​GAG​ACC​TTG​TGC​T	37 ± 112	
RAB5	CGT​AGT​CTA​GTG​TCG​ATT​AGT​TTC​CTT​GCT​ATT​GCA​GAC​CTT​GTG​CT	58 ± 14	
RAB10	TCG​AGA​GGG​ATC​TCG​GGG​CGT​GCG​ATG​ATT​TTG​CCT​TCA​T	46 ± 24	
RAB20	GCG​TTA​CGT​TAG​TGG​CCG​CCT​ATG​AGG​ACA​GGC​GGT​TGT​A	128 ± 45	
RAB28	TGG​ACG​TCG​TGG​CGG​AGG​TTT​TAT​AAA​ACG​GCG​CCA​CTG​T	49 ± 39	
RAB35	GGG​GGG​TTG​TGC​CAT​TTA​AGA​TGA​CCG​GTT​GCC​GCG​ATT​T	34 ± 5	
SA25	GGG​GAA​GGT​CGT​CCG​ACG​AAC​CCG​GTC​AGA​TAG​GGT​GGG​G	44.92 ± 1.36	Nguyen et al. (2022)
SA28	GCG​GCC​ACG​GAG​GGG​GTG​CCG​GGC​GTG​GAA​TAA​GAT​GTG​G	77 ± 1.22	
SA35	CAC​AGG​TGT​GGG​GAG​GTC​CCC​ATG​GAG​GTG​GTT​CAA​TG	58.77 ± 0.73	
SA37	AAA​GAC​GGG​GGG​GGG​GAC​CGG​CGT​ATG​AGT​GAA​GAT​GGG​G	16.5 ± 3.41	
SA40	CGA​CGT​GAA​GCA​ATC​ATG​GGT​GGG​GTA​CGT​CGG​GTC​ATG​G	126.95 ± 0.51	
SA81	AAC​GAG​GCG​CAG​GGG​GAG​GGG​GTG​GTA​CAG​ATA​AGA​TGG​GG	14.47 ± 8.18	

**TABLE 2:** Available aptasensors for detect S.aureus.

Detection methods	Strategy/Assay	Linear range (CFU/ml)	LOD (CFU/ml)	Time	References
Colorimetric	Gold nanoparticle-based colorimetric aptasensor using tyramine signal amplification (TSA) technology	10∼10^6^	9	1∼2 h	Yuan et al. (2014b)
Colorimetric	Visual detection based on aptamer recognition coupled to tyramine signal amplification	10∼10^7^	8	1∼2 h	Yuan et al. (2014a)
Colorimetric	A colorimetric based on Cu-MOF-catalyzed chromogenic reaction with aptamer recognition and magnetic separation	50∼10^4^	20	∼1 h	Wang et al. (2017a)
Colorimetric	A chemiluminescence biosensor based on nicking enzyme amplification reaction and rolling circle amplification	5∼10^4^	5	∼2 h	Xu et al. (2018)
Colorimetric	A colorimetric biosensor based on specific aptamer and catalysis of dsDNA-SYBR Green I (SG I) complex	10^2^∼10^7^	81	5∼6 h	Yu et al. (2020)
Colorimetric	A novel colorimetric immunoassay based on a combination of immunomagnetic separation and signal amplification *via* etching-enhanced peroxidase-like catalytic activity of gold nanoparticles (AuNPs)	10∼10^6^	10	∼1 h	Yao et al. (2020)
Colorimetric	on-site colorimetric based on aptamer-immobilized gold nanoparticles (aiGNPs)	1.5 × 10^7^∼5.3 × 10^7^	1.5 × 10^7^	∼1 h	Lim et al. (2021)
Colorimetric	One-step colorimetric based on target-induced shielding against the peroxidase mimicking activity of aptamer-functionalized gold-coated iron oxide nanocomposites	10∼10^6^	10	12 min	Zhang et al. (2021b)
Colorimetric	A colorimetry and fluorescenc dual-signal strategy based on Upconversion Nanoprobes	56∼5.6 × 10^6^	20	/	Ouyang et al. (2021a)
Colorimetric	A multicolorimetric assay based on oxidase mimicking activity of aptamer-functionalized manganese dioxide-coated ferriferrous oxide (apt-Fe_3_O_4_/MnO_2_) nanocomposites and oxTMB etching of gold nanorods (AuNRs)	10∼10^6^	10 (bare eye) and 1.2∼1.4(UV–visible spectrometry)	40 min	Zhang et al. (2021a)
Fluorescence	A sensitive luminescent bioassay based on dual-color upconversion nanoparticles (UCNPs) and aptamer-functionalized magnetic nanoparticles	10∼10^5^	8	40 min	Duan et al. (2012)
Fluorescence	Aptasensor simultaneous detection of various pathogenic bacteria based on multicolor upconversion nanoparticles (UCNPs)	50∼10^6^	25	40 min	Wu et al. (2014)
Fluorescent	A sensitive assay based on aptamer-functionalized silica magnetic nanoparticles and fluorophore loaded and nuclease resistant oligonucleotides-capped nanokeepers (mesoporous silica nanoparticles)	800∼10^4^	682	17 min	Borsa et al. (2016)
Fluorescence	A dual-excitation sensing method based on aptamer-functionalized quantum dots and upconverting nanoparticle	50∼10^6^	16	30 min	Kurt et al. (2016)
Fluorescence	A dual recognition strategy using aptamer-coated magnetic beads and antibiotic-capped gold nanoclusters	32∼10^8^	16	3∼4 h	Cheng et al. (2016)
Fluorescence	A transcription aptasensor by using a light-up RNA aptamer	10^2^∼10^6^	77	/	Sheng et al. (2019)
Fluorescent	A fluorescent detection based on a finely designed functional chimera sequence, a molecular beacon (MB), and strand displacement target recycling	80∼8 × 10^6^	39	2∼3 h	Cai et al. (2019)
Fluorescence	A highly selective platform based on aptamer-gated nano-materials	10∼10^3^	2 in buffer and 5 in blood	1 h	Pla et al. (2020)
Fluorescence	A fluorescent aptasensor based on strand displacement amplification (SDA) technology and unique self-assembled DNA hexagonal structure	7∼7 × 10^7^	1.7	∼5 h	Cai et al. (2020)
Fluorescent	A fluorescence biosensor based on a peptide-mediated immunomagnetic separation technique and an immunofluorescence quantum dot technique	10∼10^7^	5.407 in buffer, 19.9 in tap water and 10.7 in milk simulation	∼4 h	Wang et al. (2020)
Fluorescence	A colorimetry and fluorescenc dual-signal strategy based on Upconversion Nanoprobes	56∼5.6 × 10^6^	22	/	Ouyang et al. (2021a)
Fluorescence	A novel aptasensor based on aptamer-functionalized DNA−silver nanocluster nanofim	10^7^∼10^11^	/	>12 h	Yang et al. (2021a)
Fluorescent	A new fluorescence biosensor using aptamer- and vancomycin -copper nanoclusters as dual recognition strategy	10^2^∼10^8^	80	45 min	Pebdeni et al. (2021)
FRET	A multiplexed FRET-based aptamer biosensor using multicolor dyes as donors and carbon nanoparticles (CNPs) as a sole acceptor	10^2^∼10^6^	50	>3 h	Duan et al. (2016)
FRET	A dual-recognition FRET sensor based on fluorescent vancomycin−gold nanoclusters and aptamer−gold nanoparticles	20∼10^8^	10	30 min	Yu et al. (2017)
FRET	A FRET aptasensor based on self-assembled Fe_3_O_4_ and multicolor fluorescent carbon dots (CDs)	50∼10^7^	8	30 min	Cui et al. (2019)
FRET	A biosensing platform based on the binding protection effect of aptamer-cell complex	10^2^∼10^7^	64	1∼2 h	Lu et al. (2020b)
FRET	A fluorescent turn-on aptasensor based on the FRET between green carbon quantum dot and gold nanoparticle	10∼10^8^	10	∼2 h	Pebdeni et al. (2020)
FRET	A simple one-step FRET sensor based on the aptamer modified quantum dots (Aptamer-QDs) and antibiotic molecule of Teicoplanin functionalized-gold nanoparticles (Teico-AuNPs)	/	1/cell	∼2 h	Fu et al. (2020)
FRET	An efficient FRET sensor based on stimuli-responsive nanoprobe PDANSs-FAM-Apt	0∼3.5 × 10^8^	1	∼6 h	Ye et al. (2020)
FRET	A simple one-step FRET sensor based on aptamer-modified quantum dots (aptamer-QDs) w and antibiotic of teicoplanin functionalized-gold nanoparticles (Teico-AuNPs)	10∼5 × 10^8^	2 in buffer and 100 in milk	1 h	Tao et al. (2021)
FRET	A FRET aptasensor based on Aptamer-functionalized gold nanoparticles (AuNPs-aptamers) and cDNA-modified upconversion nanoparticles (UCNPs-cDNA)	47∼4.7 × 10^7^	10.7	17 min	Ouyang et al. (2021a) Ouyang et al. (2021b)
Ratiometric FRET	A dual-recognition ratiometric fluorescent nanosensor based on the blue fluorescence of novel π-rich CNPs and NIR fluorescent Apt-Van-QDs	0∼10^6^	1	30 min	Shen et al. (2020)
NSET	A one-step fluorometric strategy based on nanometal surface energy transfer (NSET) between carbon dots (CDs) and gold nanoparticles (AuNPs)	10∼10^6^	10	∼1 h	Yao et al. (2021)
Fluorescence imaging	A fluorescence microscopy imaging based on positive dielectro-phoresis (pDEP) driven on-line enrichment and aptamer-fluorescent silica nanoparticle (FNP)	50∼10^6^	93 in deionized water and 270 in spiked water	1∼2 h	Shangguan et al. (2015)
Fluorescence imaging	A quantitative fluorescence imaging platform on a smartphone based on aptamer-functionalized fluorescent magnetic nanoparticles	50∼2000	10	10 min	Shrivastava et al. (2018)
SERS	A magnetically assisted SERS biosensor based on Ag-coated magnetic nanoparticles, AgMNPs as SERS substrate and AuNR−DTNB@Ag−DTNB core−shell plasmonic NPs or DTNB-labeled inside-and-outside plasmonic NPs, DioPNPs as SERS tag	10∼10^5^	10	50 min	Wang et al. (2015)
SERS	A SERS biosensor based on sandwich structure by using gold nanoparticles and MGNPs immmoblized with aptamers	10^2^∼10^7^	35	2-3 h	Zhang et al. (2015a)
SERS	A microfluidic optical device based on SERS-encoded nanoparticles functionalized with aptamer	/	<15	10 min	Catala et al. (2016)
SERS	A label-free SERS detection based on aptamer dependent *in situ* formations of silver nanoparticles (AgNPs)	10∼10^7^	1.5	25 min	Gao et al. (2017)
SERS	Dual-recognition SERS biosensor based on vancomycin- -Au@MBA as SERS tags and aptamer-Fe_3_O_4_@Au as specific magnetic concentration and dual-SERS substrat	10∼10^7^	3	45 min	Pang et al. (2019)
SERS	Fluorescence and SERS dual-mode biosensor based on gold nanoparticle-modified polystyrene microspheres (Au/PS)	16∼1.6 × 10^5^	3	1∼2 h	Lei et al. (2020)
SERS	A SERS aptasensor based on AuNPs functionalized polydimethylsiloxane (PDMS)film	43∼4.3×10^7^	13	>2 h	Zhu et al. (2021a)
SERS	A SERS aptasensor based on artificial peroxidase enzyme regulated multiple signal amplified system	10∼10^6^	1.95	>3 h	Liu et al. (2021b)
SERS	A simple and novel biosensor based on target-induced release of signal molecules from aptamer-gated aminated mesoporous silica nanoparticles (MSNs) coupled with surface-enhanced Raman scattering (SERS) technology	47∼4.7 × 10^8^	17	13 min	Zhu et al. (2021b)
SERS	A SERS biosensor based on aptamer-facilitated gold/silver nanodimers and magnetic separation enrichment	3.2 × 10^2^∼3.2 × 10^7^	96	30 min	Ma et al. (2021)
SERS	A SERS biosensor based on the sandwich recognition of aptamer-functionalized magnetic beads and polyphenolic SERS nanotags	10^2^∼10^8^	10^2^	1∼2 h	Huang et al. (2021)
SPR	The SPR aptasensors via a polyadenine-mediated immobilization method	10^5^∼10^8^	10^6^	/	Wang et al. (2019b)
LSPR	A LSPR sensors based on aptamer at nanostructured plasmonic elements	/	10^3^	2 min	Khateb et al. (2020)
ECL	An electrochemiluminesce aptasensor based on the quenching effect of MoS_2_-PtNPs-vancomycin to S_2_O_8_^2−^/O_2_ system	1.5 × 10^2^∼1.5 × 10^8^	28	2∼3 h	Han et al. (2019)
CRET	An enhanced chemiluminescence resonance energy transfer aptasensor based on rolling circle amplification and WS_2_ nanosheet	50∼1.5×10^5^	15	1 h	Hao et al. (2017)
Potentiometric	Label-free potentiometric biosensors based on carbon nanotubes and aptamers	2.4 × 10^3^∼2.0 × 10^4^	8 × 10^2^	30 min	Zelada-Guillen et al. (2012)
Potentiometric	A potentiometric biosensor based on chemically modified graphene (transducer layer of the aptasensor) and aptamers (sensing layer)	/	1	1-2 min	Hernandez et al. (2014)
DPV	An electrochemical immunosensor based on dual-aptamer-based sandwich by using streptavidin coated magnetic beads (MB) and silver nanoparticles immmoblized with aptamers	10∼10^6^	1	30 min	Abbaspour et al. (2015)
DPV	A versatile signal-on electrochemical biosensor based on triple-helix molecular switch	30∼3 × 10^8^	8	>3 h	Cai et al. (2021a)
DPV	An electrochemical biosensor based on the electrodeposition of Cu metal−organic framework (Cu-MOF) thin film	7∼7 × 10^6^	1.9	30 min	Sun et al. (2021)
DPV	A dual signal amplification electrochemical biosensor based on a DNA walker and DNA nanoflower	60∼6 × 10^7^	9	140 min	Cai et al. (2021b)
EIS	Impedimetric aptasensor based on nanocomposite prepared from reduced graphene oxide and gold nanoparticles	10∼10^6^	10	60 min	Jia et al. (2014)
EIS	Impedimetric biosensor based on aptamer as biological recognition element	10∼10^9^	10	10 min	Reich et al. (2017)
EIS	An electrochemical aptasensor based on gold nanoparticles/carbon nanoparticles/cellulose nanofibers nano-composite (AuNPs/CNPs/CNFs) at the surface of glassy carbon electrode	12∼1.2 × 10^8^	1	30 min	Ranjbar and Shahrokhian, (2018)
Capacitance	A capacitance sensors array functionalized with aptamers	/	10	1 h	Jo et al. (2018)
Conductometric	Conductometric sensor based on magnetic analyte separation via aptamer	4.1×10^3^∼4.1×10^8^	4.0 × 10^3^	60 min	Zhang et al. (2020)
Volumetric bar-chart chip	A bacteria-detection V-Chip based on the extraordinary catalytic activity of platinum nanozyme and the aptamer-modified magnetic beads	1∼10^8^	1	1.5 h	Huang et al. (2019)
Flow cytometry	A dual-color flow cytometry assay based on aptamer recognition and fluorescent silica nanoparticles (FSiNPs)	/	150 in buffer and 760 in spiked milk	/	He et al. (2014)
Resonancelight-scattering	A biosensor combines aptamer-conjugated gold Nanoparticles and a resonance light-scattering-detection system	/	1	1.5 h	Chang et al. (2013)
Nanophotonic interferometric	A nanophotonic interferometric biosensor based on a bimodal waveguide interferometer (BiMW)	800∼1.6 × 10^5^	29	12 min	Maldonado et al. (2020)
Magnetoelastic	A magnetoelastic sensors based on an aptamer-modified magnetoelastic alloy	10∼10^11^	5	5∼6 min	Rahman et al. (2015)
Piezoelectric	A novel aptamer/graphene interdigitated gold electrode piezoelectric sensor by employing aptamer as a biological recognition element	41∼4.1 × 10^5^	41	∼1 h	Lian et al. (2015)
Lateral flow test strip	A lateral flow test strip based on the sandwich-type format using primary aptamer conjugated with gold nanoparticles (AuNPs) as the signal probe and a secondary aptamer-coated membrane as a capture probe	/	10^4^	10 min	Lu et al. (2020a)
Engineered aptasensor	A novel pathogen aptasensor swab based on functionalized nanobeads	10^2^∼10^5^	<100 (visual) and 2 (theoretically)	5 min	Raji et al. (2021)
Microfluidic biochip	Microfluidic device based on a polydimethylsiloxane (PDMS)/paper/glass hybrid and aptamer-functionalized graphene oxide (GO)	10^4^∼10^6^	800	10 min	Zuo et al. (2013)
Microfluidic chips	Paper-based microfluidic chips based on dual-aptamer-based sandwich	/	10^5^	35 min	Wang et al. (2019a)
Pressure-based biosensor	POC testing protocol based on vancomycin (Van)-functionalized platinum nano-particles (PtNPs@Van) and aptamer-coated magnetic CuFe_2_O_4_ nanoprobes dual-recognition units and the catalyzed gas-generation reaction	5∼10^4^	1	30 min	Li et al. (2019a)
PGM-based biosensor	Magnetic-aptamer biosensor based on the PGM platform and hybridization chain reaction strategy	3∼3 × 10^3^	2	>5 h	Yang et al. (2021b)

“/”: Not mentioned.

## Aptasensors Based on Optical Transduction

Optical biosensors have been widely used in the detection of *S. aureus* due to simple, fast, low cost, high sensitivity, real-time monitoring capability and label-free possibility. Optical biosensors can be classified according to transduction mechanisms such as absorption, scattering, diffraction, reflection, refraction and luminescence (photoluminescence, chemiluminescence, electrochemiluminescence or bioluminescence) (Paniel et al., 2013). Colorimetric, fluorescence, chemiluminescence, surface plasmon resonance (SPR) and surface-enhanced Raman scattering (SERS) are the most commonly used optical techniques (Rubab et al., 2018).

### Colorimetric-Based Aptasensor

Colorimetric is the simplest and oldest biosensors. It can detect the target by detecting the color change of the solution with the naked eye or a simple instrument such as a UV-Vis spectrophotometer. It has been widely used to monitor various targets due to its simplicity, stability and low cost (Wang et al., 2018). The key technology of colorimetric is the selection of catalytic enzymes and corresponding chromogenic substrates. Traditional catalytic enzymes such as horseradish peroxidase (HRP) show high substrate specificity and catalytic efficiency, and its substrates are usually 3,3',5,5'-tetramethylbenzidine (TMB) and 2,2'-azino-bis (3-ethylbenzothiazoline-6-sulfonic acid) (ABTS). Yuan et al. constructed a colorimetric-based aptasensor combining aptamer, HRP and tyramide signal amplification technology for the detection of *S. aureus* (Yuan et al., 2014a). With the help of tyramide signal amplification technology, the limit of detection (LOD) can reach to 8 CFU/ml, and the linear range is 10∼10^7^ CFU/ml (Yuan et al., 2014a). G-quadruplex (G4) is a DNase with a steric configuration, which has been widely used in biosensors due to its ability to combine with hemin to form a DNA-mimicking enzyme with catalase activity. Xu et al. established an ultrasensitive colorimetric-based aptasensor for *S. aureus* detection by using nickase amplification reaction (NEAR), rolling circle cycle amplification technology (RCA) and G4/hemin complex (Xu et al., 2018). The results showed that the established biosensor exhibited good discrimination between live and dead *S. aureus* in addition to good specificity, low detection limit (5 CFU/ml), and wide linear range (5∼10^4^ CFU/ml) (Xu et al., 2018).

Although HRP and G4 show high substrate specificity and catalytic efficiency, they also have disadvantages such as difficulty in storage, poor stability, and catalytic activity is easily affected by external conditions (such as pH). Since Woo discovered that Fe_3_O_4_ nanoparticles exhibited peroxidase activity in 2013 (Woo et al., 2013), Fe_3_O_4_ nanoparticles have received great attention due to their outstanding peroxidase-like activity, physicochemical stability, low cost, non-toxicity, and magnetic properties that can be used for enrichment and isolation of targets. Coating metal catalysts on the surface of magnetic nanoparticles to form hybrid nanocomposites can not only avoid the oxidation of Fe_3_O_4_ nanoparticles, but also significantly improve their catalytic performance (Zhang et al., 2021a; Zhang et al., 2021b). Zhang et al. synthesized a gold-coated Fe_3_O_4_ nanocomposites to construct a colorimetric-based aptasensor for one-step detection of *S. aureus* in water, urine and milk samples. The detection process does not require enrichment, separation or washing steps, and the results can be obtained by naked eye in 12 min. Under the optimal reaction conditions, the LOD is 10 CFU/ml, and the linear range is 10∼10^6^ CFU/ml (Zhang et al., 2021b). Besides Fe_3_O_4_ nanoparticles, a variety of metal nanoparticles have also been synthesized as peroxide analogs in recent years, such as CeO_2_ NPs, Cu-MOF NPs, Co_3_O_4_ NPs, ZnFe_2_O_4_NPs (Su et al., 2012; Wang et al., 2017a; Li et al., 2017b; Cheng et al., 2019).

**FIGURE 2:**
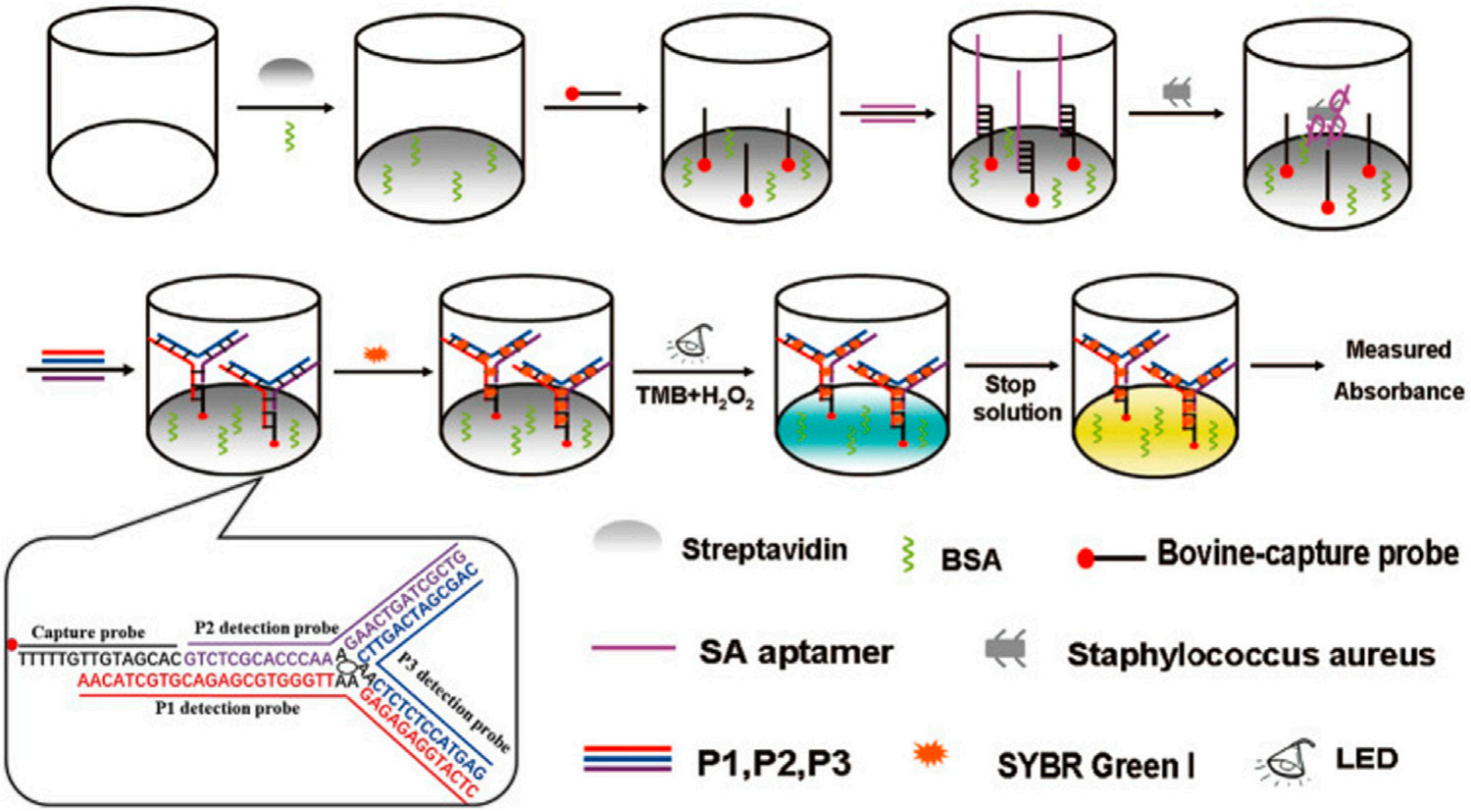
Schematic illustration of the principle for detection of *S. aureus* with aptamer-high throughput colorimetric biosensor based on photocatalytic activity of dsDNA-SG I complex. Reproduced with permission (Yu et al., 2020).

There is also a class of enzyme-free colorimetric-based aptasensors, such as gold and silver metal nanoparticles, which are widely used as signal indicators due to their size- and distance-dependent optical properties (Mokhtarzadeh et al., 2015; Taghdisi et al., 2016). The color of AuNPs solution will change from red to blue or purple when the well-dispersed AuNPs aggregate under certain conditions, which was caused by the change of the surface resonance frequency of the AuNPs. Due to the high extinction coefficient of AuNPs, the sensitivity of colorimetric sensors based on AuNPs is higher than that of organic dyes (Ghosh and Pal, 2007). Chang et al. constructed an sensor based on aptamer and AuNPs for accurate identification of *S. aureus* from common pathogens (Chang et al., 2016). In the presence of *S. aureus*, the aptamer was removed by binding to *S. aureus*. Then the AuNPs aggregated when AuNPs and high salt were added, and the color of AuNPs solution will turned to blue or purple. In the absence of *S. aureus*, the color of the AuNPs solution was red because the unscavenged aptamers can adsorbed on the surface of AuNPs and protected AuNPs from ion-induced aggregation (Chang et al., 2016). Zhang et al. discovered that the dsDNA-SYBR Green I (SG I) complex has photocatalytic activity for the first time, which can utilize dissolved oxygen to catalyze the oxidation of the substrate TMB under light irradiation (Zhang et al., 2015b). Compared with HRP and G4, dsDNA-SG I complex has obvious advantages, such as simplicity, high sensitivity and label-free. Yu et al. constructed a colorimetric-based photoirradiation aptasensor for detection of *S. aureus* based on catalysis of dsDNA-SG I complex (Figure 2). The results shown that the biosensor could specifically, sensitively, rapidly and quantitatively detect *S. aureus*. In addition, this method can be used for high throughput analysis as it can detect 96 samples at once (Yu et al., 2020).

### Fluorescence-Based Aptasensor

Fluorescence-based aptasensors mainly trigger or inactivate the fluorescence emission properties of fluorescent dyes or fluorescent nanomaterials through the interaction of aptamers with targets. Fluorescence-based aptasensors have become one of the most commonly used sensors for low-concentration analyte detection due to the advantages of the higher sensitivity, wider detection range and multiplex detection as compared with colorimetric-based aptasensors. It is relatively easy to construct distance-dependent fluorescence resonance energy transfer (FRET) aptasensors because aptamers can be easily modified with fluorophores or/and quenching dyes at the 3' or 5' end without affecting their binding affinity to the target. Traditional organic fluorescent dyes mainly include carboxyfluorescein (FAM), fluorescein isothiocyanate (FITC) and acridine orange, which have been widely used for a long time (Paniel et al., 2013). Lu et al. proposed an enzymatic cleavage aptasensor in which the aptamer act as biological recognition element for *S. aureus* in complex samples. The two ends of the molecular beacon were labeled with FAM (fluorophore) and BHQ1 (quencher) respectively, and can hybridize stably with aptamers. The aptamer can avoid being cleaved by exonuclease (Exonuclease I and III, Exo. I and III) when binging to *S. aureus* due to the enzymatic protection effect of aptamer-pathogen complex. The unscavenged aptamer then combined with the molecular beacon, which separated the fluorophore from the quencher, thereby restoring the fluorescence (Lu et al., 2020b). This detection system can be completed in the same tube without culture or separation, but its sensitivity (64 CFU/ml) need to be improved (Lu et al., 2020b).

**FIGURE 3:**
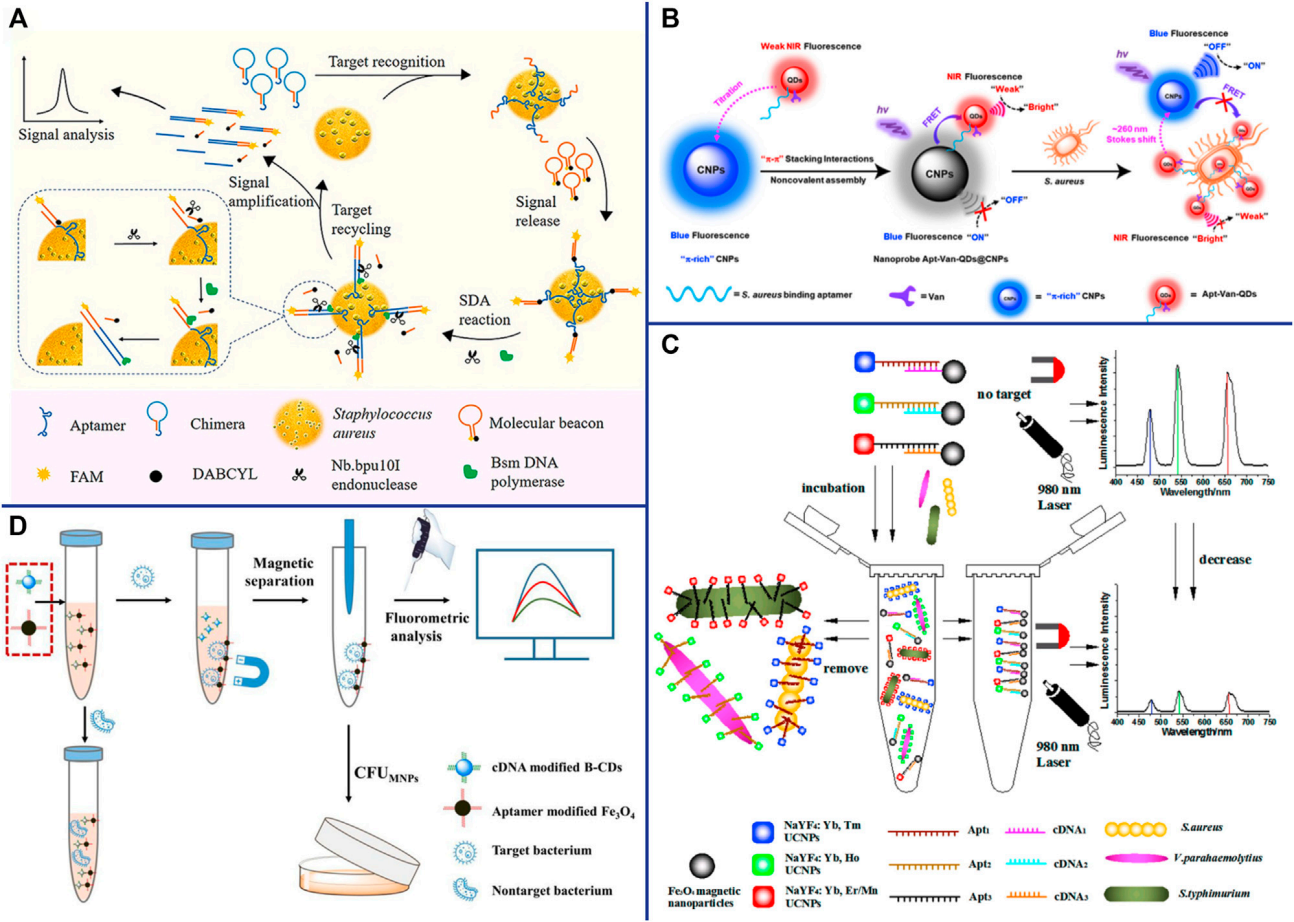
**(A)** Schematic representation of the fluorescent detection of *S. aureus* based on a finely designed functional chimera sequence, a molecular beacon (MB), and strand displacement target recycling. Reproduced with permission (Cai et al., 2019). **(B)** General design of the vancomycin and aptamer dual-recognition moieties-based ratiometric fluorescent nanoprobe with a remarkably large Stokes shift for ultrafast and accurate management of *S. aureus* at the single-cell level. Reproduced with permission (Shen et al., 2020). **(C)** Schematic illustration of the multiplexed luminescence bioassay based on aptamers-modified UCNPs for the simultaneous detection of various pathogenic bacteria. Reproduced with permission (Wu et al., 2014). **(D)** Schematic illustration of the fluorometric aptasensor for high-sensitivity *S. aureus* detection based on FRET of the self-assembled dimer, B-CD/Fe_3_O_4_. Reproduced with permission (Cui et al., 2019).

DNA amplification systems such as rolling cycle amplification (RCA), strand displacement reaction (SDA) (Cai et al., 2020), hybridization Chain reaction (HCR) (Yang et al., 2021b) and hairpin DNA cascade hybridization reaction (HD-CHR) are often used to improve the sensitivity of aptasensor. The SDA is an isothermal amplification that does not require thermal cycling, which can exponentially expand the target sequence in less than 15 min (Wang et al., 2017b). Cai et al. designed an aptasensor for *S. aureus* detection based on functional chimera sequence, molecular beacon and SDA. The functional chimera sequence contains the *S. aureus* aptamer sequence and the molecular beacon complementary sequence, and it can form a hairpin structure. The molecular beacon was labeled with FAM (fluorophore) and DABCYL (quencher) in the end respectively. The distance between FAM and DABCYL in the hairpin structure of the molecular beacon is narrow, and the fluorescence signal of FAM is quenched by DABCYL. When the aptamer in the functional chimera sequence binds to *S. aureus*, the molecular beacon complementary sequence will be exposed and bound to the molecular beacon, keeping the FAM away from the DABCYL, and thereby emitting fluorescence (Figure 3A). The established aptasensor displays good detection limit (39 CFU/ml), wide linear range (8∼8 × 10^8^ CFU/ml), satisfactory recovery and repeatability (Cai et al., 2019). However, the use of traditional organic fluorescent dyes in sensors is limited due to low fluorescence intensity and rapid photobleaching. Compared with traditional organic fluorescent dyes, fluorescent nanomaterials such as quantum dots (QDs) (Fu et al., 2020; Tao et al., 2021), upconversion nanoparticles (UCNPs) (Wu et al., 2014; Ouyang et al., 2021b) and carbon dots (Cui et al., 2019; Pebdeni et al., 2020) that have emerged in recent years have many advantages, such as wider absorption spectra, size-tunable narrower emission spectra and better stability (Yao et al., 2014).

QDs are nanoscale semiconductors (1∼20 nm in diameter), which can be effectively excited by any wavelength shorter than the emission peak and emit a narrow and symmetrical characteristic spectrum that varies with the size of the QD. Since QDs of different sizes can be excited by the same wavelength and emit different emission peaks, Wang et al. achieved the simultaneous detection of *Escherichia coli* O157:H7, *S. aureus* and *Vibrio* parahaemolyticus by modifying different aptamers with QDs of different sizes (Wang et al., 2020). Shen et al. constructed a ratiometric FRET-based aptasensor which involves target-induced emission intensity changes at two or more different wavelengths to reduces interference from various target-independent factors (Shen et al., 2020). They employed novel π-electron-rich carbon nanoparticles (CNPs) with high photostability and blue fluorescence as energy donors, and bilayer-modified QDs with aptamers and vancomycin as energy acceptors (Apt-Van-QDs). Due to the π−π stacking interaction, the Apt-Van-QDs will bind to the surface of π-rich CNPs, thereby promoting FRET between QDs and CNPs, and resulting in strong blue fluorescence quenching at ∼465 nm for the CNPs and fluorescence enhancement at ∼725 nm for Apt-Van-QDs (Shen et al., 2020). The FRET between QDs and CNPs was disrupted and exhibited a large Stokes shift of ∼260 nm when Apt-Van-QDs binds to *S. aureus* and moves away from CNPs (Figure 3B). The dual-recognition ratiometric fluorescent nanosensor showed an ultrahigh specificity and can detect a single bacteria with 30 min (Shen et al., 2020). The use of a suitable donor-acceptor is crucial to improve the efficiency of FRET. AuNPs serve as excellent energy acceptors for ultrasensitive molecular detection due to easy synthesis, good optical and colloidal stability, and high extinction coefficient. Fu et al. established a dual-recognition FRET-based aptasensor for the detection of *S. aureus* by using aptamer-modified QDs as donors and teicoplanin-modified AuNPs as acceptors. This dual-recognition FRET-based aptasensor can detect a single *S. aureus* in cells, providing a simple, specific, sensitive and rapid diagnostic method for the detection of intracellular bacteria (Fu et al., 2020). QDs are typically extracted from a mixture of lead, cadmium and silicon that are often toxic and environmentally hazardous, which limiting their applications.

Lanthanide-doped NIR-to-visible UCNPs are able to emit intense visible light upon excitation by NIR (usually 980 nm), which is not absorbed by biological samples and does not cause autofluorescence and light scattering background (Wang et al., 2010; Cheng et al., 2013b). The optical properties of UCNPs can be tuned by changing their lanthanide dopants (including Er^3+^, Tm^3+^, and Ho^3+^) (Liu et al., 2013). Wu et al. constructed a sensitive and specific biosensor based on multicolor UCNPs as luminescent labels and aptamers as biological recognition elements for simultaneous detection of *S. aureus*, *Vibrio* parahaemolyticus and *Salmonella typhimurium* (Figure 3C). Under the optimal reaction conditions, the LOD of the sensor was 25 CFU/ml and the linear range was 50∼10^6^ CFU/ml (Wu et al., 2014). AuNPs are considered as efficient fluorescence quenchers due to their large surface-to-volume ratio and strong surface plasmon absorption in the NIR and IR (Zhu et al., 2010). Taking advantage of the high fluorescence quenching effect of AuNPs, Ouyang et al. established a more sensitive FRET-based aptasensor for the detection of *S. aureus*. Aptamer-complementary sequence-modified UCNPs were used as the donor and aptamer-modified AuNPs were used as the acceptor. The fluorescence of the aptamer-complementary sequence-modified UCNPs were restored when it was replaced by *S. aureus* and released from the aptamer-modified AuNPs. The sensitivity of this detection system was 10.7 CFU/ml, and the linear range was 47∼4.7 × 10^7^ CFU/ml (Ouyang et al., 2021b). UCNPs have been widely used in sensors due to their advantages of high quantum yield, narrow emission peak, good photostability, long fluorescence lifetime, large anti-Stokes shift, and resistance to photobleaching. However, there are still some limitations, for example, it is difficult for the naked eye to distinguish different target concentrations under excitation by a 980 nm laser beam.

CDs are spherical carbon particles (or graphite fragments) luminescent materials smaller than 10 nm with semiconductor quantum effect and up-conversion function. CDs have been widely used in FRET-based sensors due to their outstanding properties, such as easy synthesis, low cost, strong fluorescence emission, high water solubility, good biocompatibility, and resistance to photobleaching. Cui et al. synthesized multi-color fluorescent CDs with long fluorescence lifetime and high photostability for *S. aureus* detection. The aptamer complementary sequences modified CDs were used as energy donors, and their fluorescence signals could be quenched by the aptamer-modified Fe_3_O_4_ through FRET. The fluorescence of the aptamer complementary sequences modified CDs were restored when they were replaced by *S. aureus* and released from the aptamer-modified Fe_3_O_4_ (Figure 3D) (Cui et al., 2019). Their result shown that the sensor has fast detection speed (<30 min), good sensitivity (8 CFU/ml) and wide linear range (50∼10^7^ CFU/ml). What’s more, CDs can be used as fluorescent probes for *in vitro* and *in vivo* bioimaging due to their excellent biocompatibility and low toxicity, providing a new platform for pathogen bioimaging detection (Cui et al., 2019). Yao et al. constructed an enzyme-free and label-free nano-metal surface energy transfer (NSET) aptasensor based on CDs (donor) and AuNPs (acceptor) for one-step detection of *S. aureus* (Yao et al., 2021). In this study, linker DNA was used to connect aptamer-modified CDs and aptamer-complementary sequence-modified AuNPs. Compared with the direct hybridization of CDs and AuNPs, the fluorescence of CDs was quenched by AuNPs as high as 63.5% in linker DNA based hybridization, which significantly improved the detection limit of *S. aureus* (Yao et al., 2021).

**FIGURE 4:**
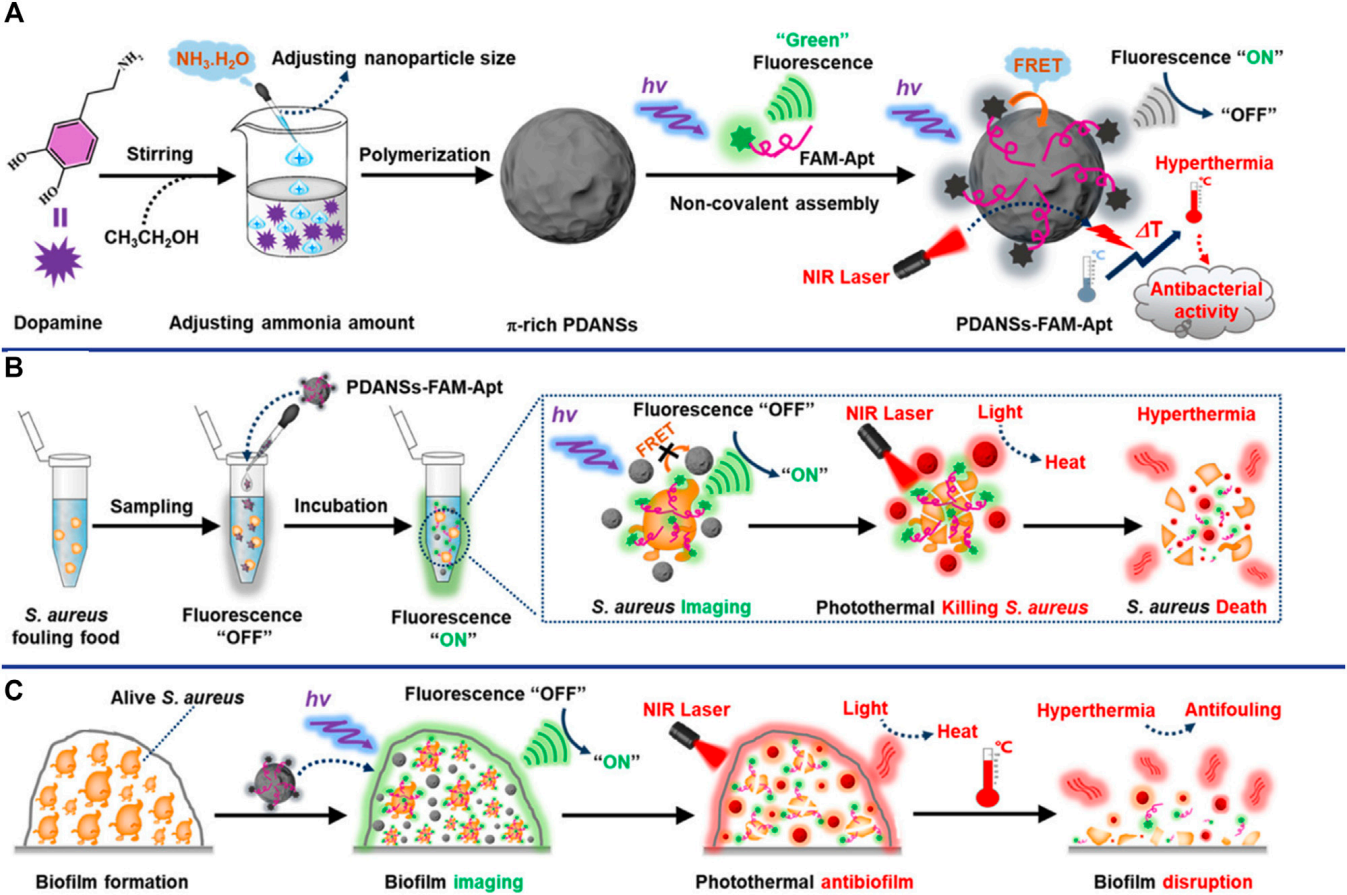
General design of the smart nanoprobe PDANSs-FAM-Apt for accurate fluorescence detection and imaging-guided precise photothermal antibacteria. **(A)** Illustration of the assembly procedure of the nanoprobe PDANSs-FAM-Apt. **(B)** Diagram of the FRET-based assay procedure for PDANSs-FAM-Apt responsive to living *S. aureus* and imaging-guided photothermal killing of *S. aureus*. **(C)** Schematic of imaging-guided photothermal antifouling of the nanoprobe PDANSs-FAM-Apt for the destruction of *S. aureus* biofilms. Reproduced with permission (Ye et al., 2020).

Nanomaterials are playing an increasingly important role in improving sensors for the detection of bacterial pathogens due to their unique optical properties, and are considered as one of the most promising candidates for accurate reporting of pathogens. Novel metal nanoclusters (NCs) are a new type of nanoparticles with diameters less than 2 nm, and are composed of less than 150 metal atoms. It has attracted more and more attention due to its outstanding advantages such as water solubility, high fluorescence and quantum yield, remarkable catalytic behavior, good biocompatibility and stability, etc (Munoz-Bustos et al., 2017; Yu et al., 2017; Pebdeni et al., 2021). Pebdeni et al. proposed a sensitive and specific *S. aureus* detection sensor based on vancomycin and aptamer dual receptor functionalized copper nanoclusters (CuNCs). CuNCs have photoluminescence properties and can induce fluorescence signal enhancement during the aggregation which was caused by *S. aureus*. Their result shown that the selectivity and sensitivity of this fluorescence sensor was enhanced by aptamer-CuNCs (Pebdeni et al., 2021). NCs not only possess unique fluorescent properties, but also have received extensive attention for their antibacterial properties (Javani et al., 2016). Yang et al. achieve a novel approach for visual detection and effective elimination of *S. aureus* by combing DNA-templated silver nanoclusters (DNA-AgNCs) and aptamers (Yang et al., 2021a). Compared with general fluorescent nanomaterials, polymer-based fluorescent nanomaterials exhibit excellent biocompatibility and biodegradability, and are easily prepared by self-polymerization (Zhang et al., 2015c). Polydopamine nanospheres (PDANS) have been used as excellent energy receptors for the construction of aptamer-based FRET biosensors because of its tunable diameters, broad absorption bands, and surface-conjugated rigid planar structures that facilitate PDANS binding interactions with aptamers *via* π-π stacking (Ye et al., 2020). Ye et al. designed a stimuli-responsive nanoprobe (PDANSs-FAM-Apt) for the detection of *S. aureus* at single-cell level, and it was able to destroy *S. aureus* and its biofilm on demand *via* NIR light-activated photothermal activity (Figure 4) (Ye et al., 2020). Porous materials with “molecular gates” have also been widely used in sensors for biomolecule detection. Nanoporous anodic aluminum (NAA) is widely used in aptasensors due to its stability, non-degradation in aqueous solution, multiple reuses after calcination, and support of nucleic acids (DNA, RNA, or aptamers) as molecular gates (Chen et al., 2015; Ribes and Aznar, 2019; Pla et al., 2020). Pla et al. installed the fluorescent indicator rhodamine B in the NAA well, and the entrance of the well was covered by DNA aptamer. The fluorescent indicator rhodamine B will be released when the orifice was opened by *S. aureus* binding to aptamer (Pla et al., 2020). This nanodevice can specific and sensitive (2 and 5 CFU in buffer and blood, respectively) detection of *S. aureus* in less than 1 h (Pla et al., 2020).

### Surface Enhanced Raman Scattering-Based Aptasensor

**FIGURE 5:**
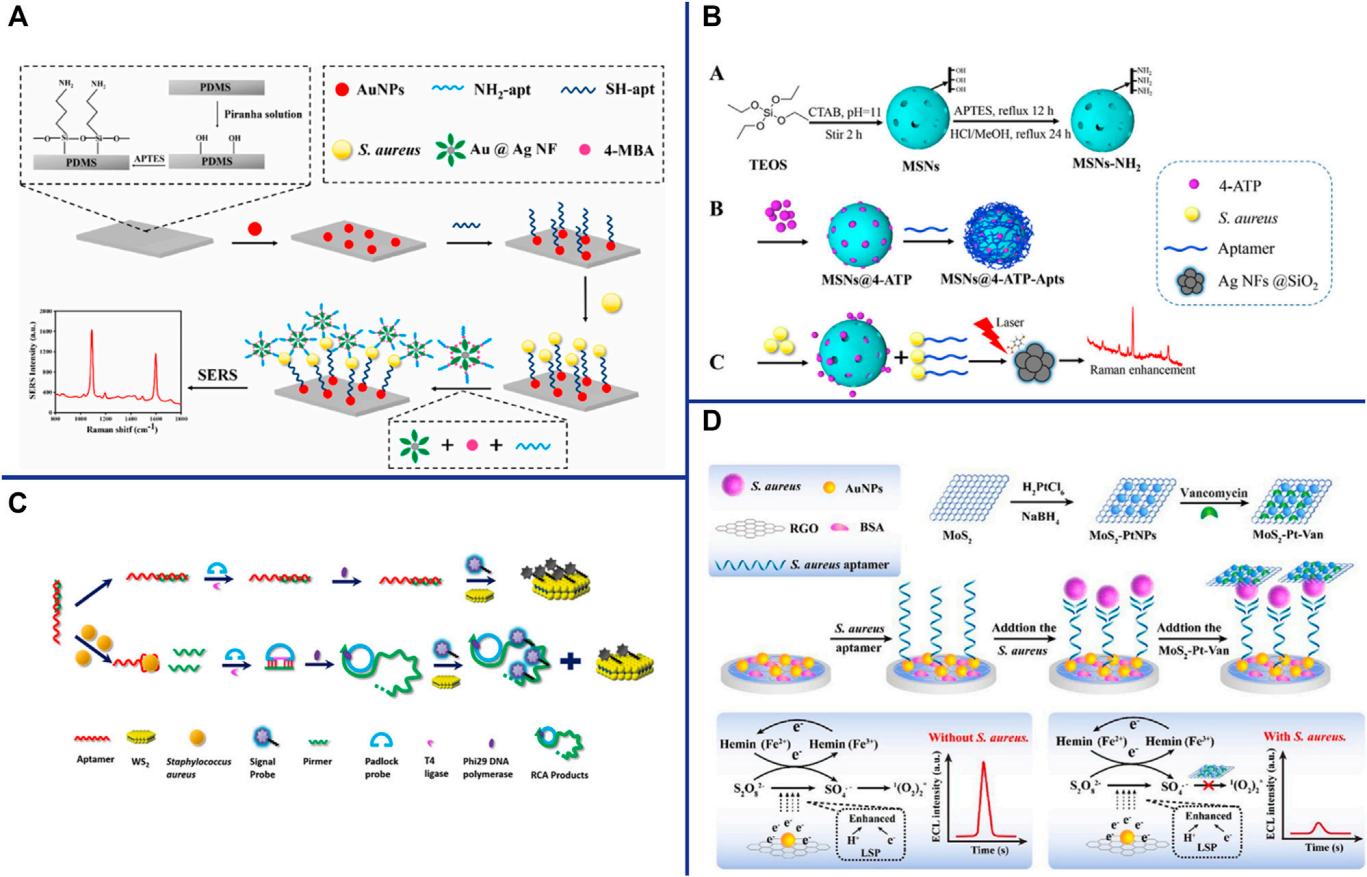
**(A)** Schematic illustration of the developed SERS biosensor based on aptamer functionalized PDMS film for the detection of *S. aureus*. Reproduced with permission (Zhu et al., 2021a). **(B)** Schematic illustration of *S. aureus* detection based on the target-responsive release of 4-ATP molecules from aptamer-gated MSNs. Reproduced with permission (Zhu et al., 2021b). **(C)** Schematic illustration of the CRET biosensor for the detection of *S. aureus* based on Co^2+^/ABEI-AuNFs and WS_2_ nanosheet. Reproduced with permission (Hao et al., 2017). **(D)** Schematic illustration of the enzyme-free ECL aptasensor for *S. aureus* detection based on AuNPs/hemin as the regenerable enhancers of S_2_O_8_^2−^/O_2_ and the quenching effect of MoS_2_-PtNPs on S_2_O_8_^2−^/O_2_. Reproduced with permission (Han et al., 2019).

“Raman scattering” means that the molecules in the substance absorb part of the energy from excitation light and vibrate in different ways and degrees (for example: the wobbling and twisting of atoms, the wobbling and vibration of chemical bonds), and then scatter lower frequency light. The frequency of Raman scattering is determined by the characteristics of the substance. The Raman scattering can be enhanced by several orders of magnitude when different molecules or ions are adsorbed on the surface of metal nanomaterials (Ag, Cu, Au and other metal nanomaterials), which is related to the submicroscopic surface roughness of metal nanomaterials. SERS-based aptasensors are widely used in pathogen detection due to their ultra-sensitive, simple and rapid, excellent photostability, label-free and non-destructive fingerprint recognition (Xu et al., 2019). Zhang et al. constructed a “capture probe-target-signal probe” sandwich sensor for *S. aureus* detection by using Raman molecules and aptamer-modified AuNPs as signal probes, and aptamer-modified Fe_3_O_4_ magnetic gold nanoparticles (MGNPs) as capture probes (Zhang et al., 2015a). Although this method is simple, rapid, wide linearity (10^2^∼10^7^ CFU/ml), high sensitivity (35 CFU/ml) and strong specificity, it is time-consuming (2∼3 h) (Zhang et al., 2015a). Gao et al. constructed a label-free SERS-based aptasensor with only 25 min for the detection of *S. aureus* (Gao et al., 2017). Using the aptamer that specifically recognizes and binds to *S. aureus* as a template, silver nanoparticles were synthesized *in situ* to directly obtain the SERS fingerprint spectrum (Gao et al., 2017). This strategy is simple, rapid and low-cost, creating an idea for *in situ* detection of pathogens on microarrays. SERS-based sensors are generally used in liquid systems, which greatly limit their application due to the large number of detection steps and high cost. Zhu et al. construct a reliable, rapid, sensitive and specific biosensor for *S. aureus* detection by using aptamer-AuNPs-polydimethylsiloxane (PDMS) as capture substrate, mercaptobenzoic acid (4-MBA) and aptamer-modified gold-silver Core-shell nanoflowers (Au@Ag NFs) as signal probes (Figure 5A) (Zhu et al., 2021a). The SERS spectra will change when the sandwich structure “capture substrate-*S. aureus*-signal probe” was formed in the presence of the *S. aureus*. The SERS aptasensor has high sensitivity (13 CFU/ml), and exhibits a good linear relationship in the range of 4.3 × 10 CFU/ml∼4.3 × 10^7^ CFU/ml. Au and Ag are the most popular SERS substrates, but their applications are limited due to their easy aggregation, high chemical activity, and easy chemical reaction with external substances (Jiao et al., 2021). Core-shell structures have recently been widely used as SERS substrates due to thin shells composed of inert materials can successfully prevent noble metal cores from contacting the external environment. Silica is an ideal shell material because it has no absorbance in the visible wavelength band and is somewhat hydrophilic (Li et al., 2017a). Zhu et al. proposed a SERS sensor for the detection of *S. aureus*, which based on *S. aureus* can induce release of the Raman dye 4-aminothiophenol (4-ATP) from mesoporous silica nanoparticles (MSNs) by binding to the gating aptamer (Figure 5B) (Zhu et al., 2021b). The SERS sensor is low-cost, fast, high sensitivity (17 CFU/ml), wide linearity (4.7 × 10 CFU/ml-4.7 × 10^8^ CFU/ml), and is more reliable for detection of foodborne pathogens (Zhu et al., 2021b). The nanodimer and nanotrimer are also widely used as SERS substrates and show a greater increase in SERS signal compared to monodisperse particles (Ma et al., 2021).

### Surface Plasmon Resonance-Based Aptasensor

Surface plasmon resonance (SPR) is a physical phenomenon that can cause resonance due to the light energy absorption of free electrons when incident light irradiates on the surface of plasmonic elements. The SPR was enhanced when light is irradiated onto plasmonic nanoparticles (eg, gold, silver, copper) or nanostructured and was called localized surface plasmon resonances (LSPRs) (Csáki et al., 2018). LSPR-based aptasensors have the advantages of ultrasensitive, real-time monitoring, label-free, miniaturization, and portability (Hoa et al., 2007). The LSPR frequency and strength primarily depends on the size, shape, geometry, interparticle spacing, and dielectric properties of the material. AuNPs have been widely used in LSPR-based aptasensors due to their easy preparation, high density, large dielectric constant, and good biocompatibility. Khateb et al. successfully constructed a simple, portable, and label-free LSPR-based aptasensor for *S. aureus* detection with short detection times of 120s (Khateb et al., 2020). Their results showed that the thickness of the sensing layer was critical, with a significantly greater responses for the thinner aptamer layer compared to antibody-based recognition elements (Khateb et al., 2020). They also measured the optical response experimentally through finite-difference time-domain simulations of differently sized metal nanostructures (100 and 200 nm disks) based sensor, and showed that the sensitivity of the 200 nm diameter disk structure was significantly higher compared to the 100 nm diameter disk structure, which is due to the increase in bulk refractive index sensitivity and the extent to which the local field extends from the metal surface (Khateb et al., 2020). The LSPR can also be simply tuned by changing the shape of the NPs, for example, only one LSPR has occurred in the 520 nm range (red color) for gold nanospheres (2∼50 nm), while two were detected in the range of 522 nm (transverse LSPR) and 698 nm (longitudinal LSPR) for gold nanorods (Sharifi et al., 2020).

### Chemiluminescence-Based Aptasensor

Chemiluminescence (CL)-based biosensors are a promising detection method because their energy is generated by chemical reactions and the sample does not require photoexcitation, thus avoiding interference from light scattering, unstable light source, and high background (Dodeigne et al., 2000). Hao et al. constructed a Chemiluminescence resonance energy transfer-based aptasensor for *S. aureus* detection using Co^2+^-enhanced N-(aminobutyl) N-(ethylisoluminol) (ABEI)-modified AuNFs (Co^2+^/ABEI-AuNFs) as the donor and WS_2_ nanosheets as the acceptor (Figure 5C). The limits of detection of the electrochemiluminescence (ECL)-based aptasensor was 15 CFU/ml with the help of RCA amplification technology (Hao et al., 2017). Han et al. constructed an ECL aptasensor for *S. aureus* detection based on AuNPs/hemin as a regenerable enhancer for S_2_O_8_^2−^/O_2_ and MoS_2_-PtNPs as a quencher for S_2_O_8_^2−^/O_2_ (Figure 5D) (Han et al., 2019). RGO was coated on the GCE to increase the specific surface area and electronic conductivity. The thiol-modified aptamers were then immobilized on the AuNPs which were electrochemically deposited on the RGO surface. In the presence of *S. aureus*, the signal was turned off because aptamer-*S. aureus* -MoS_2_-PtNP-Van sandwich complex formed which act as quencher for S_2_O_8_^2−^/O_2_. Conversely, in the absence of *S. aureus*, the signal was turned on (Han et al., 2019). The results shown that the ECL-based aptasensor has high specificity and sensitivity (28 CFU/ml), and can be used for the detection of *S. aureus* in urine samples (Han et al., 2019).

## Aptasensors Based on Electrochemical Transduction

The electrochemical aptasensors refers to that when the aptamer immobilized on the electrode surface binds to the target, it will cause a change that can be converted into a measurable electrical signal (current, impedance, potential or conductance, etc.) (Li et al., 2019b). Electrochemical sensors are one of the preferred biosensors for their high sensitivity, low cost, multi-analyte analysis, reproducibility, miniaturization and portability (Maduraiveeran et al., 2018). Electrochemical sensors can be classified into potentiometric, amperometric/voltammetric, impedimetric and conductometric sensors according to the electrical signal measured (Paniel et al., 2013).

### Potentiometric-Based Aptasensor

Potentiometric biosensor is one of the oldest electrochemical techniques and is widely used due to its low cost and simple operation. Recent studies have shown that an increasing number of electrochemical sensors employ nanomaterials as efficient transducers and amplifiers. Carbon nanotubes have been widely used as transducers in electrochemical sensors due to their strong ability to promote electron transfer between electroactive species and electrodes. Zelada-Guillén et al. construct a highly sensitive potentiometric sensor for real-time detection of *S. aureus* without labeling based on aptamers as biorecognition elements and single-walled carbon nanotubes (SWCNTs) as potential transducers (Zelada-Guillen et al., 2012). The results shown that the sensor could detect S. aureus in skin in real time with good stability and specificity (Zelada-Guillen et al., 2012). Graphene oxide (GO) and reduced graphene oxide (RGO) are the preferred material for fabricating highly sensitive sensing platforms due to their unique thermal, mechanical, and electrical properties. Hernández et al. constructed a potentiometric aptasensor based on GO and RGO for the detection of S. aureus. The potentiometric aptasensor has strong selectivity and high sensitivity and can detect single live *S. aureus*. However, when the potentiometric aptasensor was applied to real samples, it is necessary to filter out the electroactive substances in the sample that interfere with the detection (Hernandez et al., 2014).

### Amperometric/Voltammetric-Based Aptasensor

**FIGURE 6:**
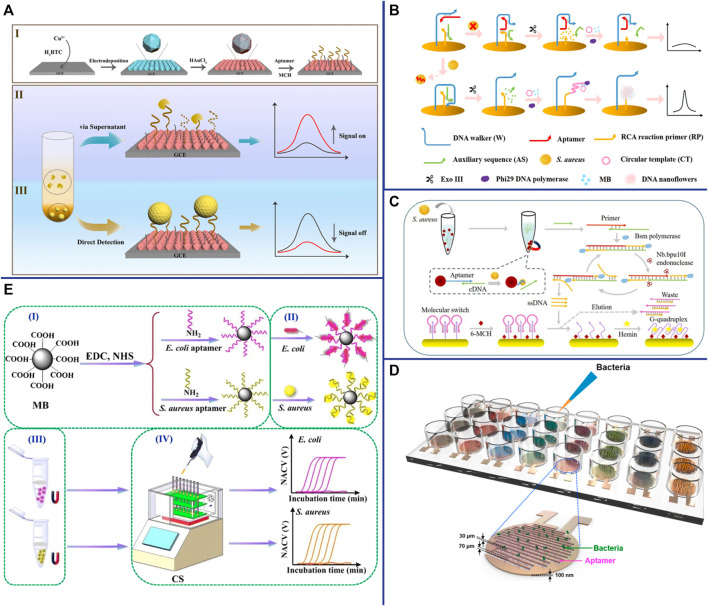
**(A)** (I) Fabrication of the DNA/AuNPs/MOFs-based sensing platform; (II) Schematic diagrams of the electrochemical biosensor for the detection of *S. aureus* via supernatants; and (III) pathogen cells. Reproduced with permission (Sun et al., 2021). **(B)** Schematic representation of the biosensor for *S. aureus* detection based on a DNA walker and DNA nanoflower. Reproduced with permission (Cai et al., 2021b). **(C)** Schematic representation of the versatile signal-on electrochemical biosensor for *S. aureus* detection based on triple-helix molecular switch. Reproduced with permission (Cai et al., 2021a). **(D)** Schematic of aptamer-functionalized capacitance sensor array for real-time monitoring of bacterial growth and antibiotic susceptibility. Reproduced with permission (Jo et al., 2018). **(E)** Schematic representation of the multichannel conductometric sensor for *S. aureus* detection based on magnetic analyte separation *via* aptamer. (I)the preparation of aptamer-functionalized magnetic beads, (II) selective capture and (III) separation of bacterial cells, and (IV) determination of viable bacteria by the conductometric sensor. Reproduced with permission (Zhang et al., 2020).

Amperometric/voltammetric is the most common and successful electrochemical techniques for *S. aureus* detection. Abbaspour et al. constructed a voltammetric-based aptasensor for *S. aureus* detection, which based on aptamer-modified magnetic beads as capture probes and aptamer-modified silver nanoparticles (Apt-AgNPs) as signal probes. Apt/*S. aureus*/apt-AgNP sandwich complexes form in the presence of *S. aureus*, resulting in current changes (Abbaspour et al., 2015). This approach with good sensitivity (1.0 CFU/ml) and wild linear range (10∼10^6^ CFU/ml) due to the combination of magnetic bead separation and signal amplification of AgNPs (Abbaspour et al., 2015). Besides metallic nanomaterials, other nanomaterials as efficient amplifiers have also become an integral part of electrochemical biosensor. For example, metal-organic framework (MOF) is a unique inorganic-organic hybrid nanomaterial, which has been widely used in many fields due to its flexibility, uniform structure, good chemical stability, and good biocompatibility (Han et al., 2020). Sun et al. electrodeposited Cu metal-organic framework (Cu-MOF) thin films on electrodes and *in situ* reduced AuNPs on their surfaces to enhance their electrical conductivity and form complexes (DNA/AuNPs/MOFs) with aptamers *via* Au-S bonds to construct electrochemical biosensors for dual detection of *S. aureus* (Figure 6A) (Sun et al., 2021). This aptasensor not only has high specificity and high sensitivity (1.9 CFU/ml), but also is easy to operate and requires no additional signal components (Sun et al., 2021). With the development of DNA nanotechnology, various nanomaterials constructed from DNA have also been widely used in electrochemical sensors. Cai et al. constructed a dual-signal amplifying electrochemical aptasensor based on DNA walker and DNA nanoflowers for high-sensitivity detection of *S. aureus* (Cai et al., 2021b). Two sets of double-stranded DNA were modified on the surface of the Au electrode. When the aptamer was bound to *S. aureus*, the DNA walker bound to the aptamer was released. With the help of exonuclease III (Exo III), the DNA walker moves along the electrode surface and continuously hydrolyzes the anchored short double-stranded DNA. The introduction of circular DNA and phi29 DNA polymerase initiates RCA and in turn forms nanoflowers, which provide binding sites for electroactive methylene blue (MB), resulting in a strong electrochemical signal (Figure 6B) (Cai et al., 2021b). The results shown that the electrochemical aptasensor has high sensitivity, high specificity and good anti-interference ability, which can distinguish *S. aureus* in mixed samples from other non-target bacteria (Cai et al., 2021b). In addition to nanomaterials, nucleic acid-based RCA, SDR, HCR, and HD-CHR amplification systems are also widely used as amplifiers in electrochemical sensors. Cai et al. constructed a multifunctional electrochemical sensor based on SDA and triple helix molecular switch for *S. aureus* detection (Cai et al., 2021a). In the presence of *S. aureus*, the aptamer binds to *S. aureus* and releases the complementary sequence (cDNA) into the solution, which initiates the SDA reaction under the action of primers and enzymes, and resulting in a large number of ssDNA probes. Subsequently, the interaction of the ssDNA probe with the triple helix molecular switch on the electrode leads to the release of the G-quadruplex-rich capture sequence, which reacts with hemin to form an electroactive G-quadruplex/heme complex and then turns on the electrochemical signal (Figure 6C) (Cai et al., 2021a). The results shown that this aptasensor has high sensitivity (8 CFU/ml), high specificity, and can detect *S. aureus* in lake water, tap water and honey samples (Cai et al., 2021a).

### Impedimetric-Based Aptasensor

In recent years, impedimetric-based aptasensor has attracted interest for the detection of *S. aureus* due to its high sensitivity, no matrix interference, simple measurement, no labeling, and automation (Paniel et al., 2013). Jia et al. constructed an sensitive, specific and label-free impedimetric-based aptasensor for rapid and quantitative detection of *S. aureus* (Jia et al., 2014). This system adopts a novel nanocomposite material (rGO-ssDNA-AuNPs) that enhances electron transfer and electrochemical signals as an amplifier to improve the detection sensitivity (10 CFU/ml) (Jia et al., 2014). An effective strategy to improve the performance of biosensors is to increase the reaction surface area and thus capture the target more effectively. In recent years, cellulose nanofibers (CNFs) have been widely used in biosensing due to their large surface area, high aspect ratio, extensive chemical modification ability, good biocompatibility and adhesion (Golmohammadi et al., 2017). Ranjbar et al. using carbon nanoparticles (CNPs) and AuNPs to overcome the poor electrical conductivity of CNFs to construct nanocomposites (AuNPs/CNPs/CNFs) with large surface area, excellent electrical conductivity and good biocompatibility for constructing impedimetric aptasensor (Ranjbar and Shahrokhian, 2018). Studies have shown that the aptasensor has high sensitivity (1 CFU/ml), wide linear range (1.2 × 10∼1.2 × 10^8^ CFU/ml), and can be applied to the accurate detection of *S. aureus* in human serum samples (Ranjbar and Shahrokhian, 2018). Electrochemical aptasensor not only used for *S. aureus* detection, but also be used for rapid antibiotic susceptibility testing (AST) of *S. aureus*. The aptamers were immobilized between the two finger electrodes of an interdigitated gold electrode (IDE). When *S. aureus* was captured by the aptamer, it can act as a dielectric particle connecting electrode, thereby increasing the capacitance (Figure 6D) (Jo et al., 2018). The result shown that this aptamer-functionalized capacitive sensor array can not only rapidly and specifically recognize *S. aureus* (1 h), but also monitor bacterial growth in real time with high sensitivity (10 CFU/ml), which can be used for AST of *S. aureus* (Jo et al., 2018).

### Conductometric-Based Aptasensor

Conductometric is a measurement of monitoring the conductivity of solution using a low-amplitude alternating current potential, which relies on the change in conductivity that occurs in the sample through the production or consumption of charged species. The working electrode must be in contact with the solution in traditional electrochemical sensors, which inevitably causes electrode degradation and non-specific capture. To solve these problems, Zhang et al. developed a conductometric for real-time quantifying the number of live bacteria by using aptamer-modified magnetic beads to separate *S. aureus* and then a capacitively-coupled non-contact conductivity detector to measure and record conductivity changes. The bacteria captured by aptamer-modified magnetic beads can also be used for AST (Figure 6E) (Zhang et al., 2020).

## Aptasensor For POCT

Point-of-care testing (POCT) refers to a miniature mobile detection system that is close to the test sample in a non-laboratory environment and reports the results quickly. Although POCT does not require tedious operating steps, professional operators, and expensive and sophisticated instruments, the development and popularization of highly sensitive and specific POCT technology is still a major challenge in the field of science and engineering. A major strategy of POCT is to infiltrate liquid reagents into filter paper and various water-absorbing materials, and fix them on rigid substrates after drying. Paper-based lateral flow strips (LFS), also known as test strips, have been widely used for POCT and are the most promising method for POCT in various fields. In addition to being sensitive and specific, LFS is simple to operate and does not require complicated and expensive instruments, and the detection results can be observed with the naked eye. Lu et al. constructed an aptamer- and AuNPs-based LFS for rapid detection of *S. aureus*, with a detection time of only 10 min and no cross-reaction with other common bacteria (Lu et al., 2020a). Raji et al. developed a novel aptamer-based swab sensor for qualitative and quantitative detection of MRSA on environmental surfaces (Raji et al., 2021). In the presence of MRSA on environmental surfaces, MRSA can be combined with aptamers immobilized on cotton swabs and aptamers modified by blue nanobeads to form an “aptamer-MRSA-aptamer” sandwich structure (Raji et al., 2021). The swab sensor is fast (5 min), sensitive (theoretical value 2 CFU/ml), specific, and can be read directly by the naked eye without the need for laboratory equipment (Raji et al., 2021). This novel aptasensor holds great promise because of its good detection performance, ease of use and relatively inexpensive to produce.

**FIGURE 7:**
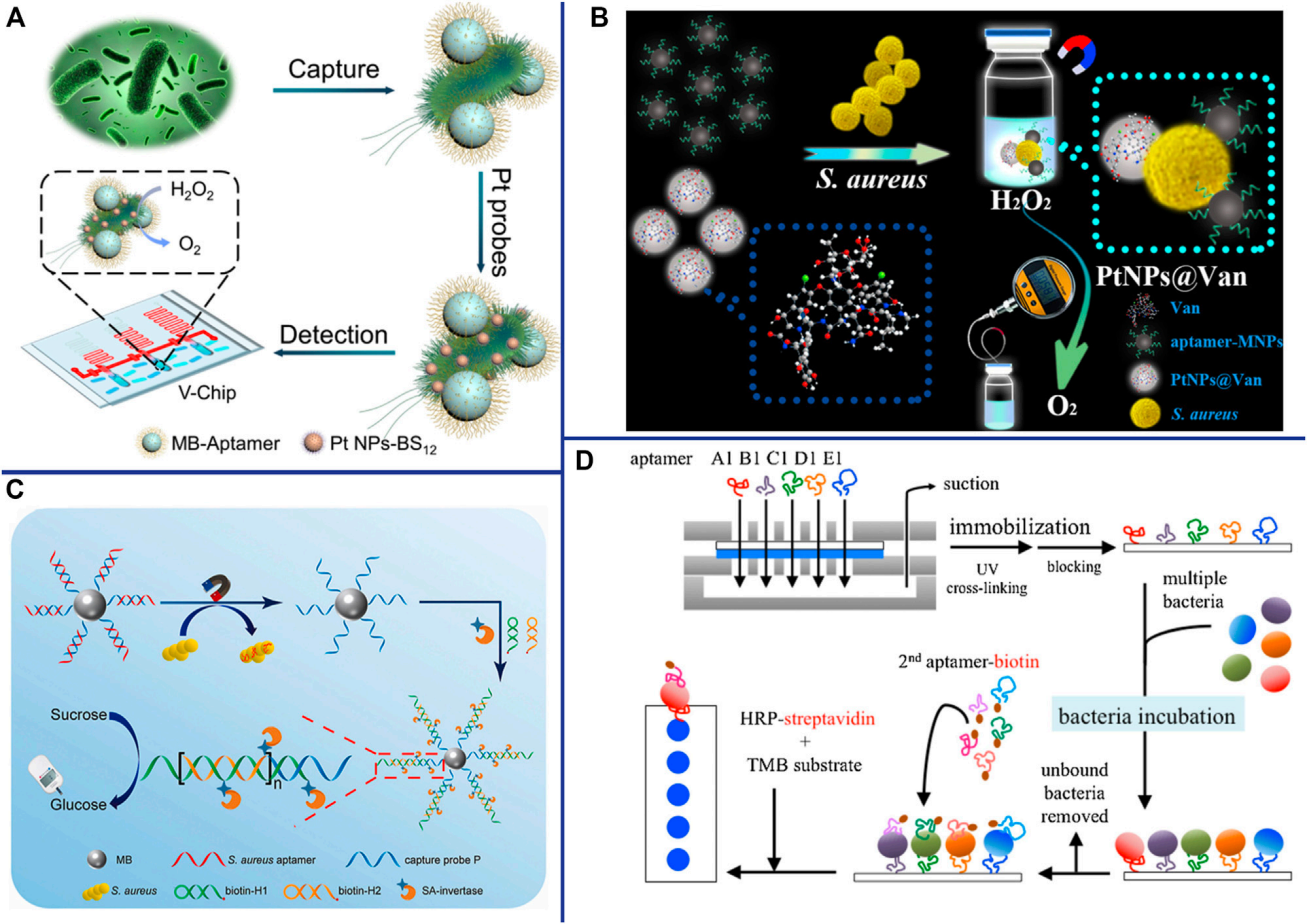
**(A)** Working principle for detection of bacteria using BV-Chip. Reproduced with permission (Huang et al., 2019). **(B)** Schematic illustration of gas pressure-based POC testing protocol for highly sensitive and specific detection of *S. aureus*. Reproduced with permission (Li J. et al., 2019a). **(C)** Schematic illustration of the principle for portable detection of *S. aureus* using PGM based on HCR strategy. Reproduced with permission (Yang et al., 2021b). **(D)** The experimental procedure for multi-bacterial detection *via* bacteria-specific aptamers immobilized on a nitrocellulose (NC) membrane. 2nd (secondary) aptamer-biotin conjugated with biotin. Reproduced with permission (Wang et al., 2019a).

Another major strategy of POCT is to miniaturize the analytical instrument and simplify the operation method, making it a portable and palm-like device. Huang et al. have developed a microfluidic device based on volumetric bar-chart chip (V-Chip) technology for rapid and visually intuitive quantification of pathogenic bacteria in urine (Huang et al., 2019). Bacteria can combine with aptamer-modified magnetic beads and 2-(dodecyldimethylamino) acetate (BS-12) modified platinum nanoparticles (PtNPs-BS12) to form an “aptamer-bacteria-PtNPs-BS12” sandwich complex. Pt NPs with extremely high catalase-like activity can catalyze the decomposition of H_2_O_2_ to generate O_2_, thereby pushing the pink ink in the V-Chip into the S-shaped readout channel (Figure 7A) (Huang et al., 2019). Due to the fast detection speed (1.5 h) and high sensitivity (as low as 1 CFU/ml) of the device, and the detection results of clinical urine samples are consistent with the plate counting, the V-Chip can significantly improve the early diagnosis rate of bacterial infection (Huang et al., 2019). A pressure measuring instrument is an ideal POCT that is simple, portable and capable of quantitative detection. Li et al. constructed a pressure-based POCT for the detection of *S. aureus* using vancomycin-modified functionalized platinum nanoparticles (PtNPs@Van) and aptamer-modified magnetic CuFe_2_O_4_ nanoprobes. The Pt NPs catalyzed the decomposition of H_2_O_2_ to O_2_ in a sealed device, resulting in a significant increase in pressure that can be detected with a hand-held digital pressure gauge (Figure 7B) (Li et al., 2019a). Personal Glucose Meters (PGMs) also has been widely used as a readout means for highly sensitive non-glucose target identification and quantification due to its advantages of portability, speed, low cost, easy operation and reliable results. Yang et al. designed a novel aptasensor for *S. aureus* detection based on HCR and PGMs (Figure 7C). This aptasensor is specific, sensitive (2 CFU/ml) and accurate, providing a new perspective for the development of sensitive and portable pathogen detection solution in the future (Yang et al., 2021b). Wang et al. developed an aptamer-based micro-microfluidic chip for multi-pathogen detection, replacing the traditional single-pathogen detection (Figure 7D). The results shown that this new microfluidic chip has faster detection time (35 min) and smaller size (7.0 cm × 5.0 cm × 1.2 cm), higher specificity, and simultaneous detected multiple pathogens compared with traditional chips (Wang et al., 2019a). Therefore, it promises to be a powerful POCT for multibacterial diagnosis.

## Conclusion and Perspectives

This review presented an overview on the latest progress of aptasensors based on optical (colorimetric, fluorescence, SPR and SERS) and electrochemical (potentiometric, voltammetric, impedimetric and conductometric) for *S. aureus* detection. Overall, colorimetric-based aptasensors are simplicity, fast, low-cost, and the results can be quickly judged by the naked eye or simple instruments. However, its low sensitivity still needs to be improved when applying to practical detection. Fluorescence-based aptasensors have become one of the most commonly used sensors for low-concentration analyte detection due to their high sensitivity, but their results are susceptible to interference by autofluorescence or background fluorescence, and the fluorescent molecules used are unstable and prone to photobleaching. Novel optical transduction mechanisms such as SPR and SERS have also been used for *S. aureus* detection due to their advantages of ultra-sensitivity, real-time monitoring, and label-freeness, but their widespread application is limited by cumbersome sample preparation and the expensive equipment required. Compared with optical based aptasensors, electrochemical based aptasensors demonstrate the potential for the fabrication of POCT due to their fastness, simplicity, sensitivity, miniaturization and portability.

Although the development of aptasensors is the most active research topic in recent years, there are still some significant challenges in practical applications: First, since most of the existing aptamers are screened by time-consuming and inefficient traditional SELEX, it is necessary to introduce new technologies and new methods (such as Cell-SELEX, Genomic SELEX, IP-SELEX, Capture-SELEX, CE-SELX, M-SELEX, AFM-SELEX, AEGIS-SELEX, Animal-SELEX) to improve the screening efficiency and generate higher affinity and specificity aptamers. Second, aptamer density, aptamer orientation, surface charge and steric hindrance during the progress of aptamer immobilization should also be systematically studied to further improve aptamer properties and biostability. Although nanomaterials play an important role in increasing the immobilization density and orientation of aptamers, high-density immobilization will limit the formation of normal configurations of aptamers. Third, although most studies have shown that the aptasensors have good specificity and could distinguish other co-existing foodborne pathogens in artificially contaminated samples (recoveries ranged from 85.0 to 110.0%), the interference effects of various metal ions, anions and antioxidants that may be present in the real sample also require further studies to ensure the existing aptasensors compatibility in complex biological and clinical samples. Fourth, the stability and reproducibility of nanomaterials that play an increasingly important role in aptasensors still need to be improved. Fifth, how to achieve simultaneous detection of multiple pathogens is still a major challenge in a short period of time.

Overall, although there are still some challenges to be solved, aptasensors have great potential in the field of *S. aureus* detection. Rapid, sensitive, specific, high-throughput, simple operation, small sample size, low cost, reagent-safe, portable, and wearable aptamer based novel microfluidic chip will become the focus of future biosensor research, and will have a significant impact on human health and well-being.
